# Ultra-processed foods and plant-based alternatives impair nutritional quality of omnivorous and plant-forward dietary patterns in college students

**DOI:** 10.1038/s41598-025-88578-0

**Published:** 2025-02-04

**Authors:** Svenja Fedde, Miriam Wießner, Franziska A Hägele, Manfred J Müller, Anja Bosy-Westphal

**Affiliations:** Institute of Human Nutrition and Food Science, Kiel University, Düsternbrooker Weg 17, 24105 Kiel, Germany

**Keywords:** Nutrition, Epidemiology

## Abstract

The health benefits of a plant-based diet may be outweighed by an increased consumption of ultra-processed foods (UPF) and plant-based alternatives. This study compares diet quality (intakes of protein, saturated fatty acids, sugar, fiber, and micronutrients) and nutritional status (prevalence of low holotranscobalamin and ferritin levels) among different dietary patterns: 22.5% vegans, 46.5% vegetarians, 31% omnivores in 142 first-year college students (20 ± 1.6 years, BMI 21.9 ± 3.1 kg/m², 83% female). Intakes of vitamin B12, folate, iron, zinc, and calcium were on average below reference values, especially in vegans and vegetarians. However, the prevalence of low holotranscobalamin and ferritin levels did not differ between the dietary groups, presumably due to supplementation. Irrespective of the diet, UPF contributed to 49% of daily energy intake. UPF exhibited a lower content of protein, fiber, vitamin B2, vitamin B12, folate, zinc and calcium compared to processed foods (all *p* < 0.001). Plant-based alternatives contained more fiber and less saturated fatty acids whereas the content of protein and micronutrients was lower compared with animal products (all *p* < 0.05). In conclusion, UPF consumption contributes to the inadequate intake of many micronutrients by young adults. This is further aggravated by plant-forward eating patterns including the consumption of plant-based alternatives.

## Introduction

Certain life transitions, such as moving from home to university, can alter established dietary patterns^1^. This can lead to changes in food intake that are mostly unfavorable, such as low consumption of fruits and vegetables and a higher intake of sugar and alcohol^2,3^. The negative shift in dietary behavior due to newfound independence accompanied by exposition to various new experiences^4^ may explain the frequently observed weight gain during the first year at university (for review, see^5^). In addition, factors such as time constraints and financial limitations^3^, as well as a lack of experience in meal planning and preparation^6^, contribute to the adoption of unhealthy eating habits, often characterized by the consumption of ultra-processed foods (UPF).

Today, UPF products constitute a major part of the food supply in retail^7^ and fast-food or takeout restaurants^8^. These products are designed to be ready-to-eat or ready-to-heat, hyper-palatable, and are affordable for most people^9^. The trend towards a higher UPF consumption was shown to be aggravated in a plant-based diet because of increased consumption of plant-based alternatives that facilitate the maintenance of traditional eating habits^10^. In line with this observation, the sales of plant-based alternatives increased rapidly during the last years^11^. In Germany, about 3% of the adult population are vegan, 9% are vegetarian, and 41% report following a flexitarian diet^12^. A plant-based diet is thereby particularly popular among younger people with a high educational level^12^ and environmental consciousness^13^ due to ethical considerations and health reasons (for review, see^14^). However, the health benefits of a plant-based diet were outweighed by an unhealthy, more processed dietary pattern^15^ that is usually characterized by a higher glycemic load and index, added sugar, and lower levels of dietary fiber, unsaturated fats, micronutrients, and antioxidants^15^. The impact of plant-based alternatives on diet quality is unclear, given the significant variation in nutritional quality among these products^16,17^. Nutritional adequacy of contemporary plant-based diets is therefore important in order to differentiate recommendations for these diets as part of a sustainable diet transformation.

The aim of the present study was to compare the dietary adequacy (intakes of protein, saturated fatty acids, sugar, fiber and micronutrients) and nutritional status (prevalence of low holotranscobalamin and ferritin levels) among different dietary patterns (omnivores, vegetarians and vegans) in first-year college students. It is hypothesized that the consumption of UPF and the subset of plant-based meat and dairy alternatives impair the nutritional adequacy of the diet.

## Methods

### Study population

The present study was conducted between November 2020 and January 2024 at the Institute of Human Nutrition and Food Science at Kiel University, Germany. The study’s primary aim was to assess changes in body weight in first-year college students (freshman weight gain). The study population is a random sample that was not intended to be representative of all first-year students in Kiel (> 5,000 students per year). First-year students from all universities in Kiel were recruited through notice board postings and social media platforms at the beginning of their first semester. A web-based recruitment questionnaire was used to determine whether the first-year students met the inclusion criteria (first semester at university, ages between 18 and 25, vegan, vegetarian, or omnivore diet ≥ 3 months). Exclusion criteria were regular medication use, chronic diseases (especially bowel diseases), and electrical implants (due to the manufacturer’s recommendations of bioelectrical impedance analysis). Afterwards, a personal interview was conducted at the institute to verify the information. Eligible individuals were subsequently invited for the examination visit, where body composition was examined, and blood sampling took place, followed by a 2-week period in which the diet was recorded on three consecutive days. 151 participants were examined, 142 of whom provided complete dietary records. Self-reported eating habits were used to classify participants comprising vegans, vegetarians, and omnivores. Written informed consent was obtained from each participant. The study protocol was approved by the medical ethics committee of Kiel University, Germany (AZ D 534/20) and followed the guidelines based on the ‘Declaration of Helsinki’. The trial was registered at ClinicalTrials.gov as NCT04598022.

### Body composition

Height was determined without shoes using a stadiometer (SECA, Modell 285, Hamburg, Germany). Body weight was measured on a calibrated scale, and body composition was assessed using bioelectrical impedance analysis (mBCA 515, seca GmbH & co. kg., Hamburg, Germany), both in underwear after an overnight fast. Fat mass index (FMI) and fat-free mass index (FFMI) were calculated as fat mass or fat-free mass divided by height squared (kg/m^2^).

### Blood sampling

Blood samples were collected after an overnight fast (≥ 10 h) between 06:30 a.m. and 10:00 a.m. Vitamin B12 status was assessed by analyzing serum holotranscobalamin (HTC) immunologically via a chemiluminescence microparticle assay. Iron status was assessed by analyzing serum ferritin using an immunoturbidimetric method. The analysis of all blood parameters was performed in an accredited and certified laboratory (Labor Dr. Krause & Kollegen MVZ GmbH, Kiel, Germany).

### Lifestyle data

A study-specific online questionnaire was used to collect sociodemographic data, including sex, age upon study participation, duration and reason for the choice of dietary pattern, smoking status, income (including the share of food expenditure), and whether they used dietary supplements (particularly vitamin B12 and iron supplements).

### Dietary data

Participants self-recorded their dietary habits using dietary records for three consecutive days, including one weekend day. They were instructed to document the following information for all consumed food products and beverages: time and place of consumption (to assess out-of-home consumption), brand and product name, as well as preparation instructions. All food quantities were weighted or estimated by the participants in grams or household measures. Daily macro- and micronutrient intakes were calculated using the nutrition software PRODI® 6.11 (Nutri-Science GmbH, Freiburg, Germany). The German Nutrient Database (BLS, Bundeslebensmittelschlüssel) Version 3.02 was used to evaluate unprocessed foods such as fruit and vegetables, but also for some processed foods such as bread, cheese, and canned goods. For most processed food products, recipes based on the manufacturer’s information had to be created by hand, as most were not included in the BLS. The ingredients listed in those recipes were based on information from the BLS in order to assess the nutrients of all ingredients (in particular, micronutrients). In case of incomplete product information, equivalent food products from the BLS were used or, if not available, comparable products from other manufacturers. Standardized recipes were developed for composite foods and dishes not described sufficiently to identify all ingredients and intake quantity. This applied to most takeaway or restaurant dishes and some homemade dishes without further details. The Dietary Reference Intake values of the German Society for Nutrition^18^ were used to evaluate nutrient intake. No underreporting was observed according to Willet’s criteria (> 500 kcal/d for females and > 800 kcal/d for males). This may be due to the exclusion of incomplete dietary records (< 3 days) and the intensive support provided during the protocol phase.

### Level of food processing

All reported food items were categorized according to the extent and purpose of food processing following the NOVA classification system^19^. This system differentiates between four food groups: 1. unprocessed and low-processed foods, 2. culinary ingredients, 3. processed foods, and 4. ultra-processed foods (UPF). In order to minimize errors, two researchers and one dietitian conducted a consensus-based classification process based on the recommendations of Martínez-Steele et al.^20^. Therefore, a list of all consumed food products was compiled, with single-ingredient foods classified as NOVA 1 or 2. Multi-ingredient food products that were clearly industrially manufactured were assigned to NOVA 4. Accuracy of classification was improved by using available information like the brand and product name (ingredient list), the preparation method (e.g., homemade, takeaway, restaurant), or certain key phrases in the food product description (e.g., fresh, unpackaged, ready-made, instant). For any remaining uncertainty, the food product was assigned to the most likely NOVA group based on the majority of other participants’ responses, findings from other studies, or else, the product was systematically assigned to the lower NOVA group. These food products were flagged for sensitivity analysis to calculate a lower and upper NOVA distribution range representing inter-rater agreement.

Qualitative aspects of the diet were determined by analyzing the contribution of each NOVA food group to the daily absolute and relative intake of energy, protein, SFA, sugar, and fiber, as well as vitamin B2, vitamin B12, folate, iron, zinc, and calcium. In order to compare qualitative aspects of consumed foods (food level) between NOVA food groups 3 and 4, the average content of macronutrients is given as the energy percentage of food, and the micronutrient content is given as 100 g of food. By assessing both qualitative aspects of the diet and foods, we are able to detect if a lower nutrient content of NOVA 4 foods may be compensated by a higher intake of these products, which results in an overall sufficient nutrient intake.

### Classification of plant-based alternatives

Products mimicking meat or dairy were defined as plant-based alternatives. Tofu or seitan in their original form were also included in this category as they are often used as meat alternatives in Western diets. Whenever possible, the plant-based alternatives were categorized according to NOVA based on the ingredient information. If brand information was missing, meat alternatives were classified as ultra-processed^19,20^. Plant-based milk alternatives with missing brand information were assigned to NOVA 3 following the best practice approach of Martinez-Steele et al. (77% of reported plant-based milk alternatives were not ultra-processed)^20^. Plant-based yogurt, cream, and cheese alternatives were categorized more frequently as ultra-processed, which is why NOVA 4 was chosen in cases of missing brand information.

The contribution of plant-based alternatives to qualitative aspects of the diet was calculated overall and within the different diet groups by analyzing their contribution to total dietary intake of energy, protein, SFA, sugar, and fiber, as well as vitamin B2, vitamin B12, folate, iron, zinc, and calcium. In order to compare qualitative aspects of plant-based alternative products (categorized into meat alternatives and dairy alternatives) with qualitative aspects of milk and dairy products, the average content of macronutrients is given as energy percentage of food, and the micronutrient content is given as 100 g of food.

### Statistical analysis

Statistical analyses were conducted using IBM SPSS Statistics^©^ (SPSS 28.0, Inc., Chicago, IL, USA) with the significance level set at p < 0.05. Normal distribution of data was rejected using the Kolmogorov–Smirnov test, and the data are presented as medians and interquartile ranges. Differences between diet groups regarding basal characteristics, daily intake of macro- and micronutrients, blood parameters, as well as the contribution of plant-based alternatives to qualitative aspects of the diet were examined using the Kruskal–Wallis test with the Bonferroni post-hoc test for metric variables and chi-square test with pairwise comparison for categorical variables. Daily intake of various micronutrients was compared between tertiles of UPF consumption, and p-values were obtained using the Kruskal–Wallis test with the Bonferroni post-hoc test. Spearman’s correlation coefficients were computed to determine the impact of UPF consumption on BMI and FMI. To assess qualitative differences between NOVA 3 and NOVA 4 food products, an unpaired two-sided Welch t-test was conducted due to large group sizes. These data are presented as mean ± SD. Graphs were plotted using GraphPad Prism 10.2.2 (GraphPad Prism for Windows, GraphPad Software, La Jolla, California, USA).

## Results

Main characteristics of the study participants overall and stratified into vegans, vegetarians, and omnivores are shown in Table 1. 83% of participants were female, with no differences between diet groups regarding age, percentage of smokers, or monthly expenditure on food. Omnivores maintained their dietary habits since childhood, while vegans and vegetarians have only been eating their diet for a median of 2–3 years (p < 0.001).

**Table 1:** Main characteristics of the study population.

	Total (n = 142)	Vegans (n = 32)	Vegetarians (n = 66)	Omnivores (n = 44)	p-value*
Males, n (%)	24 (16.9)	3 (9.4)	12 (18.2)	9 (20.5)	0.414
Females, n (%)	118 (83.1)	29 (90.6)	54 (81.2)	35 (79.5)	
Age, years	20 (19–21)	20 (19–21)	20 (19–20)	20 (19–21)	0.511
BMI, kg/m^2^	22 (20.9–23.4)	20.6^a^ (18.9–23.0)	21.3^a^ (20–22)	22.2^b^ (21–24.3)	0.005
FMI, kg FM/m^2^	f: 6.0 (5.0–7.1) m: 2.5 (2.1–4.1)	f: 5.3 (4.6–7.1) m: 3.1 (1.3–4.8)	f: 5.9 (5.1–7.0) m: 2.5 (1.9–4.3)	f: 6.5 (5.6–8.1) m: 2.5 (2.3–3.8)	f: 0.112 m:0.942
Cigarette smoker, n (%)	20 (14.1)	6 (18.8)	6 (9.1)	8 (18.2)	0.280
Food Expenditure, €/month	150 (100–200)	160 (100–250)	150 (100–200)	140 (90–213)	0.268
Adherence to diet, years	5 (2–19)	2^a^ (1–3.5)	3^b^ (2–7)	20^c^ (19–21)	< 0.001
Consumption of vitamin B12 supplements, n (%)	44 (31.0)	25^a^ (78.1)	15^b^ (22.7)	4^b^ (9.1)	< 0.001
Consumption of iron supplements, n (%)	25 (17.6)	12^a^ (37.5)	12^b^ (18.2)	1^c^ (2.3)	< 0.001

Data presented as median (interquartile range) unless otherwise stated; * p-values refer to differences between diet groups using chi-square with pairwise comparison and Kruskal–Wallis test with Bonferroni post-hoc test; ^a,b^ diet groups not sharing a common superscript letter are significantly different, p < 0.05; BMI, body mass index; f, female; m, male.

### Comparison of the diet quality and nutritional status between three diet groups

Vegans and vegetarians had a lower BMI (p = 0.005) and a tendency towards a lower energy intake (p = 0.06; Table 2) compared to omnivores. 60% of participants reported taking dietary supplements, with a particularly high intake among vegans, who also had a higher regular intake of vitamin B12 and iron supplements compared with vegetarians and omnivores (Table 1; p < 0.001). The three diet groups showed typical nutritional patterns, with vegans consuming less protein, less SFA, and more fiber than omnivores (Table 2). Vegans also had a lower vitamin B2 and calcium intake than vegetarians and omnivores while having the highest folate intake (all p < 0.01).

**Table 2:** Nutritional adequacy of omnivorous and plant-forward dietary patterns (daily dietary intake of macro-, micronutrients and salt in vegans, vegetarians and omnivores reported in 3d-dietary protocols, without supplements).

	Total (n = 142)	Vegans (n = 32)	Vegetarians (n = 66)	Omnivores (n = 44)	p-value*	% above or below dietary recommendations** Vegans, Vegetarians, Omnivores
Energy [kcal]	2050 (1722–2381)	1842 (1525–2245)	2104 (1829–2440)	2028 (1729–2442)	0.063	
Protein [E%]	12.8 (11.5–15.3)	11.9^a^ (9.6–14.8)	12.5^a^ (11.1–14.2)	14.7^b^ (12.9–16.9)	< 0.001	
Fat [E%]	34.0 (29.5–39.2)	30.0^a^ (26.0–35.8)	35.5^b^ (30.8–40.1)	33.8^a.b^ (29.7–40.1)	0.013	25, 55, 43
SFA [E%]	12.2 (9.1–15.7)	8.6^a^ (6.3–10.5)	12.7^b^ (9.9–16.9)	14.5^b^ (10.6–16.1)	< 0.001	34, 76, 84
MUFAs [E%]	11.7 (9.8–13.4)	11.1 (9.2–12.7)	12.2 (10.7–13.6)	11.3 (9.5–14.2)	0.205	
PUFAs [E%]	6.4 (5.2–8.4)	8.1^a^ (5.7–10.1)	6.8^a.b^ (5.3–8.5)	5.8^b^ (4.8–6.7)	< 0.001	75, 91, 95
Carbohydrates [E%]	46.8 (43.0–50.6)	49.6^a^ (46.6–51.7)	46.0^a.b^ (43.2–49.5)	45.1^b^ (40.2–48.7)	0.016	
Total sugar [E%]	15.6 (12.3–20.1)	16.1 (14.1–20.3)	15.4 (12.2–20.5)	15.4 (11.9–19.5)	0.593	91, 86, 86
Fiber [E%]	2.8 (2.2–3.6)	3.9^a^ (3.1–4.9)	2.8^b^ (2.2–3.4)	2.3^c^ (1.9–2.8)	< 0.001	
Salt [g/d]	5.2 (3.6–6.7)	5.0 (3.3–6.1)	5.4 (3.5–6.7)	5.2 (3.9–7.5)	0.320	25, 38, 43
Vitamin B2 [mg/d]	1.1 (0.9–1.5)	0.8^a^ (0.7–1.1)	1.2^b^ (0.9–1.5)	1.2^b^ (0.9–1.7)	< 0.001	72, 41, 36
Folate [µg/d]	276.7 (211.8–368.2)	364.5^a^ (247.7–456.0)	288.7^a.b^ (217.9–346.6)	227.2^b^ (174.8–316.4)	0.001	41, 55, 73
Zinc [mg/d]	9.8 (7.8–12.1)	8.9 (6.5–11.3)	9.8 (7.9–12.0)	10.6 (8.6–12.5)	0.086	31, 18, 14
Calcium [mg/d]	771 (558–1050)	552^a^ (484–681)	914^b^ (655–1151)	791^b^ (553–1064)	< 0.001	97, 61, 70

Data presented as median (interquartile range); *p-values refer to differences between diet groups using Kruskal–Wallis test with Bonferroni post-hoc test; ^a,b^ diet groups not sharing a common superscript letter are significantly different, p < 0.05; E%, energy percent; MUFA, monounsaturated fatty acids; PUFA, polyunsaturated fatty acids; SFA, saturated fatty acids. ** > 35% fat, > 10% SFA, < 10% PUFA, > 10% Sugar, > 6 g Salt, < 1.1 mg Vitamin B2, < 300 µg Folate, < 7 mg Zinc, < 1000 mg Calcium (according to dietary reference intake values of the German Society for Nutrition^18^.

**Fig. 1:**
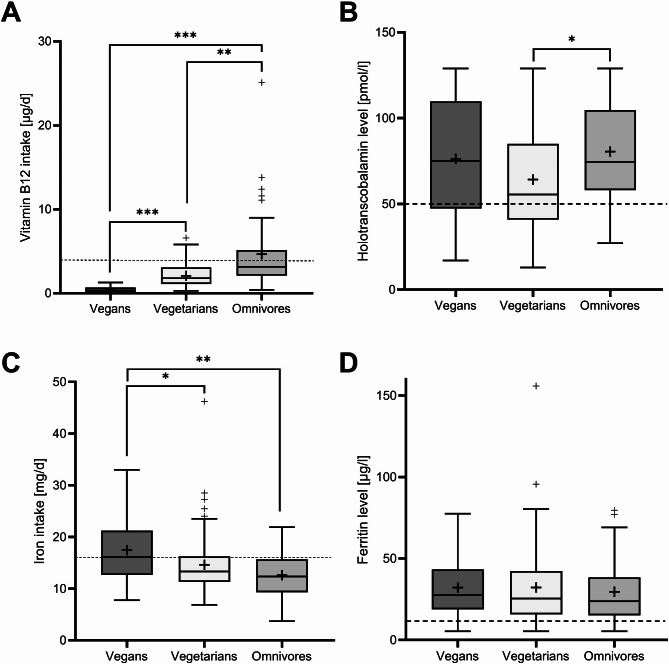
Comparison of daily vitamin B12 intake (**A**) and Holotranscobalamin levels (**B**) as well as daily iron intake (**C**) and Ferritin levels (in females, **D**), all differentiated by diet groups; --- reference values: vitamin B12 intake (according to the German Society for Nutrition): 4 µg/d, holotranscobalamin (according to laboratory specifications): 50 pmol/l, iron intake (according to the German Society for Nutrition): 16 mg/d (female), ferritin (according to laboratory specifications): 15 µg/l (female); p-values from Kruskal–Wallis test with Bonferroni post-hoc test; *p < 0.05, **p < 0.01, ***p < 0.001.

According to the reference value of vitamin B12 intake from the German Society for Nutrition (4 µg/d), 84% of participants had an inadequate vitamin B12 intake without considering supplement intake. Vegans had a lower vitamin B12 intake than vegetarians and omnivores (p < 0.001; Fig. 1A). Using HTC as a marker of B12 status, lower serum concentrations were observed in vegetarians than in omnivores (p < 0.05; Fig. 1B). The prevalence of low HTC levels was 27% overall, with 25% among vegans, 35% among vegetarians, and 16% among omnivores. Notably, females accounted for 97% of the HTC levels that were too low. A correlation between vitamin B12 intake and HTC levels was observed in omnivores only (r = 0.414, p = 0.005).

Vegans were found to have a higher iron intake (excl. supplements) than vegetarians and omnivores (Fig. 1C). 38% of vegans, 53% of vegetarians, and 68% of omnivores were found to have an insufficient iron intake according to the reference value of iron intake from the German Society for Nutrition (16 mg/d in females). However, based on ferritin levels, 80% of participants showed an adequate iron status, with no differences in ferritin concentrations between diet groups for both females (Fig. 1D) and males (data not shown; all p > 0.05). No correlation was found between iron intake and iron status, even when the data were stratified by sex and diet group (all p > 0.05).

### Qualitative aspects of UPF and their contribution to nutritional quality of the diet

A total of 8,626 foods (incl. beverages) were consumed over the 3-day protocol phase. According to the best practice approach from Martinez-Steele et al.^20^, all foods were categorized into the four NOVA categories. UPF contributed to 49% of daily energy intake (Table 3), with 5.9% of foods that could not be assigned with certainty to NOVA categories, resulting in a lower and upper bound of 45.5 – 53.0% energy intake from UPF. UPF accounted for 46.2%, 50.5%, and 48.7% of daily energy intake in vegans, vegetarians, and omnivores, respectively (p = 0.514). Because no differences between diet groups were observed, the contribution of UPF to qualitative aspects of the diet was analyzed for all participants combined. The most common UPFs in omnivorous and plant-forward eating patterns were bakery products, followed by fast food and convenience foods, sweets and desserts, and sauces and spreads.

**Table 3:** Contribution of NOVA food groups to qualitative aspects of the diet (protein, saturated fatty acids, sugar, and fiber) in first-year students (*n* = 142), and mean protein, saturated fatty acids, sugar, and fiber content of consumed foods according to each NOVA group.

	Mean energy intake		Mean protein intake		Mean protein content [E% of food]	Mean saturated fat (SFA) intake		Mean SFA content [E% of food]	Mean sugar intake		Mean sugar content [E% of food]	Mean fiber intake		Mean fiber content [E% of food]
	kcal/d	E%	g/d	%		g/d	%		g/d	%		g/d	%	
NOVA 1 (*n* = 4100)	519 ± 317	25.5	19.7 ± 13.7	27.8	12.3 ± 14.0	2.8 ± 2.8	10.9	3.1 ± 7.3	25.6 ± 16.8	31.2	30.2 ± 31.3	12.3 ± 8.0	39.4	5.5 ± 6.5
NOVA 2 (*n* = 461)	87 ± 88	4.2	0.1 ± 0.6	0.2	0.9 ± 3.0	2.7 ± 4.6	8.3	21.9 ± 26.6	1.8 ± 3.9	2.0	21.5 ± 38.1	0.0 ± 0.3	0.2	0.3 ± 1.4
NOVA 3^#^ (*n* = 1496)	431 ± 260	21.3	18.8 ± 13.4	26.5	19.0 ± 15.3	7.5 ± 6.5	25.8	14.4 ± 18.0	13.4 ± 11.3	16.8	15.3 ± 22.2	6.4 ± 5.7	20.5	3.7 ± 3.9
NOVA 4^#^ (*n* = 2569)	1020 ± 472	49.0	32.1 ± 16.9	45.5	13.1 ± 16.0***	16.0 ± 9.8	54.9	13.4 ± 14.7	42.1 ± 29.9	50.0	22.3 ± 27.6***	10.9 ± 5.4	39.9	2.6 ± 10.9***

Data presented as mean ± SD; ^#^tested for differences between mean protein, saturated fatty acids, sugar and fiber content of processed (NOVA3) and ultra-processed (NOVA 4) foods with unpaired two-sided Welch t-tests; ***p < 0.001; E%, energy percent.

A higher intake of UPF correlated with FMI in females (r = 0.22; p = 0.017) but not in males. There was no association between UPF intake and BMI in both sexes (p > 0.05).

Table 3 shows that UPF accounted for a larger intake of energy% of protein, SFA, sugar, and fiber compared to NOVA 1–3. The average protein content of UPF was, however, 6% lower than in processed foods (NOVA 3; p < 0.001). Compared with NOVA 3, UPF had a 1% lower fiber and a 7% higher sugar content, whereas there were no differences in SFA content between these two NOVA categories. In addition, UPF had a higher energy density than processed foods from NOVA 3 (2.88 vs. 1.95 kcal/g; p < 0.001).

**Fig. 2:**
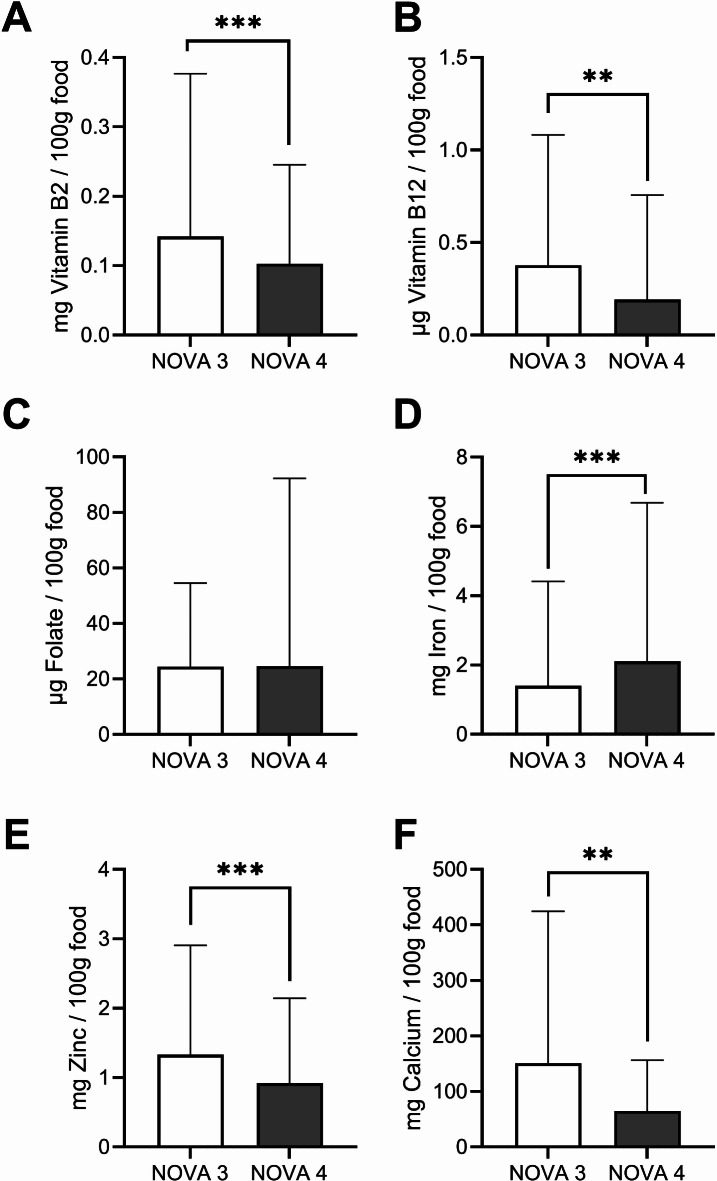
Mean Vitamin B2 (**A**), Vitamin B12 (**B**), Folate (**C**), Iron (**D**), Zinc (**E**) and Calcium (**F**) content of consumed processed (NOVA3, *n* = 1496) and ultra-processed (NOVA 4, *n* = 2569) foods from first-year students. All data are expressed as mean ± SD; p-values from unpaired two-sided Welch t-tests; **p < 0.01, ***p < 0.001.

Although UPF contributed to the largest proportion of intake of critical micronutrients for plant-based diets among all NOVA categories (vitamin B2: 40%, vitamin B12: 53%, iron: 47%, zinc: 39% and calcium: 36%), on the food level, UPF contained 50% less vitamin B2, 29% less vitamin B12, 31% less zinc and 57% less calcium than processed foods per 100 g food (NOVA 3, Fig. 2). On the contrary, iron content in UPF was 50% higher, primarily due to plant-based foods with high cocoa content, soy-based products, or processed meat products. Folate intake was mainly obtained (42%) from unprocessed foods (NOVA 1), with no differences in folate content between UPF and processed foods (NOVA 3).

Comparing vitamin B12 and iron intake between tertiles of UPF consumption (tertile 1: 14.0%–40.8% energy from UPF; tertile 2: 40.8%–56.1% energy from UPF; tertile 3: 56.1%–86.0% energy from UPF) no differences were observed regarding iron intake (p > 0.05). By contrast, the highest tertile of UPF consumption showed a higher intake of vitamin B12 compared to the lowest tertile (2.5 vs. 1.1 µg/d, p = 0.014). The main sources of vitamin B12 among UPF were meat products, fortified plant-based alternatives, fortified juices, soft drinks (energy drinks), and energy/protein bars.

### Qualitative aspects of plant-based meat and dairy alternatives and their contribution to nutritional quality of the diet

Plant-based dairy alternatives, compared with dairy products, had a lower content of all micronutrients (all p > 0.05) except for iron, which was higher in plant-based dairy alternatives (p < 0.001; Table 4). Plant-based meat alternatives contained more calcium, folate, and iron and less vitamin B2, vitamin B12, and zinc than meat products (all p < 0.001). Of the plant-based alternatives consumed, 17% were fortified with micronutrients (dairy alternatives: 23%; meat alternatives: 1%), primarily with vitamin B12 (12%), with an average fortification of 0.7 µg/100 g food.

**Table 4:** Mean Vitamin B2, Vitamin B12, Folate, Iron, Zinc and Calcium content of consumed plant-based dairy alternatives (*n* = 484) and milk/dairy products (*n* = 689) as well as of consumed plant-based meat alternatives (*n* = 162) and meat/ meat products (*n* = 71) from first-year students.

	B2 content (mg/ 100 g)	B12 content (µg/ 100 g)	Folate content (µg/ 100 g)	Iron content (mg/ 100 g)	Zinc content (mg/ 100 g)	Calcium content (mg/ 100 g)
Dairy Alternatives	0.06 ± 0.20	0.13 ± 0.40	11.84 ± 25.48	0.55 ± 0.45	0.25 ± 0.32	37.23 ± 49.80
Milk/Dairy products	0.22 ± 0.14***	0.86 ± 0.80***	14.73 ± 14.67*	0.18 ± 0.23***	1.54 ± 1.81***	306.58 ± 351.42***
Meat Alternatives	0.08 ± 0.08	0.01 ± 0.14	38.27 ± 56.45	2.20 ± 1.81	1.06 ± 0.90	99.80 ± 73.17
Meat /Meat products	0.17 ± 0.07***	1.30 ± 0.93***	6.32 ± 7.58***	1.33 ± 0.63***	2.01 ± 1.07***	15.34 ± 10.10***

All data are expressed as mean ± SD; p-values from unpaired two-sided Welch t-tests (comparison between plant-based dairy alternatives and milk/dairy products; comparison between plant-based meat alternatives and meat/ meat products); *p < 0.05, ***p < 0.001.

**Fig. 3:**
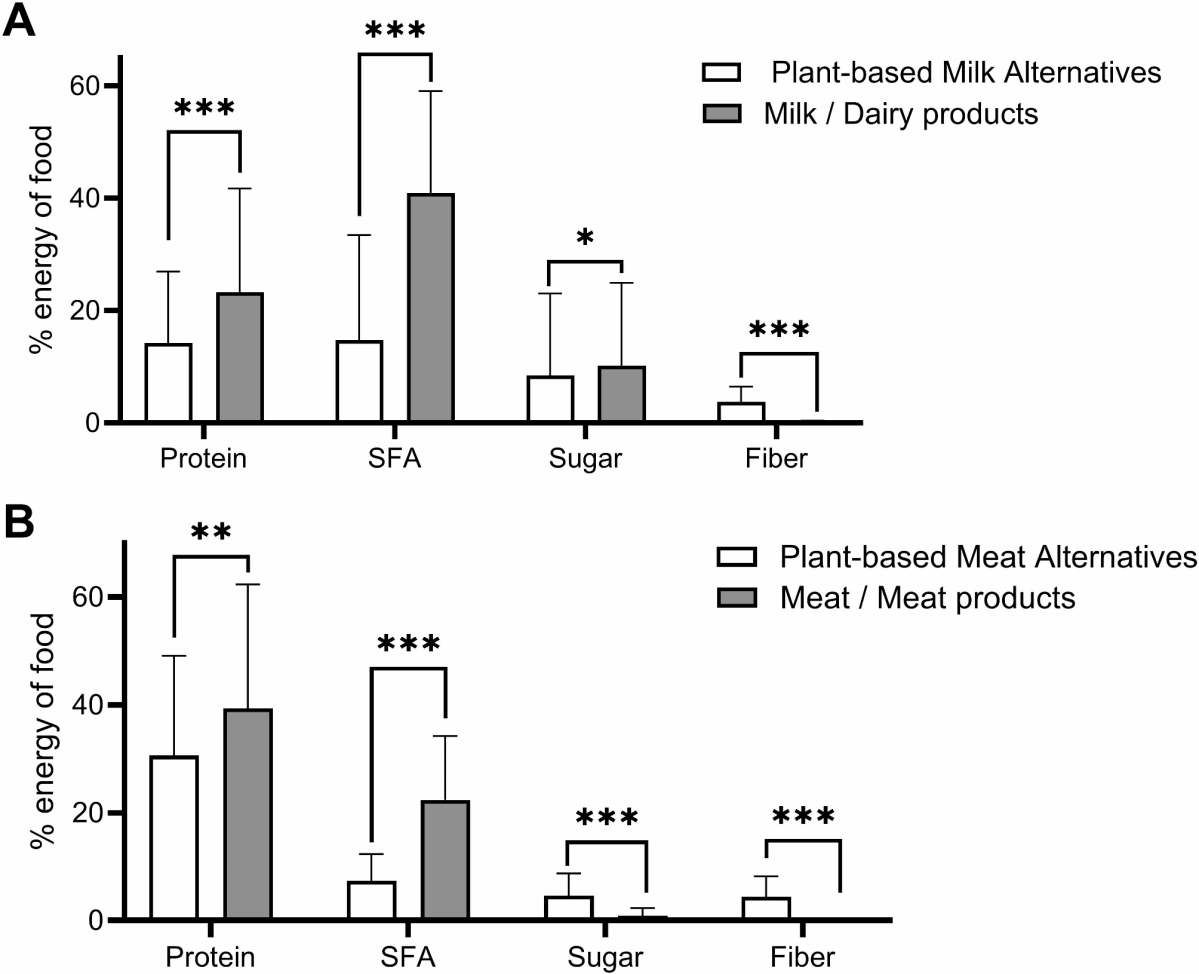
Mean content of qualitative aspects of the diet (protein, saturated fatty acids, sugar and fiber) of consumed (**A**) plant-based dairy alternatives (*n* = 484) and milk/dairy products (*n* = 689) as well as of consumed (**B**) plant-based meat alternatives (*n* = 162) and meat/ meat products (*n* = 71) from first-year students (*n* = 142). All data are expressed as mean ± SD; p-values from unpaired two-sided Welch t-tests; *p < 0.05, **p < 0.01, ***p < 0.001; SFA, saturated fatty acids.

Figure 3 compares the qualitative aspects of plant-based dairy alternatives with milk and dairy products (Fig. 3A) and plant-based meat alternatives with meat and meat products (Fig. 3B). More plant-based alternatives were categorized as UPF compared to animal-based products (dairy alternatives: 51%, dairy products: 13%; meat alternatives: 96%, meat products: 66%; both p < 0.001). The energy density of consumed plant-based meat and dairy alternatives was lower than that of animal-based products (dairy alternatives and dairy products: 1.2 ± 1.7 vs. 3.0 ± 2.2 kcal/g; meat alternatives and meat products: 1.7 ± 0.7 vs. 2.4 ± 0.9 kcal/g; both p < 0.001). Plant-based dairy alternatives contained 9% less protein, 26% less SFA, 2% less sugar, and 4% more fiber than milk and dairy products (Fig. 3A). Plant-based meat alternatives contained 8% less protein, 15% less SFA, 4% more sugar, and 4% more fiber than meat and meat products (Fig. 3B).

Plant-based alternatives were consumed by 79% of the participants, with 100% of vegans reporting daily consumption, followed by vegetarians (85%) and omnivores (55%, difference between diet groups p < 0.001). Accordingly, plant-based alternatives accounted for a larger proportion of daily energy intake in vegans (12%) compared to vegetarians and omnivores (7% and 5% of total energy intake, p < 0.001, Table 5). The percentage of protein and micronutrient intake from plant-based alternatives was at least as high as the percentage of energy intake from these foods. However, the percentage of SFA intake from plant-based alternatives was also higher than the energy intake from these foods. Although 44% of vitamin B12 intake was derived from plant-based alternatives in vegans, these products did not contribute to vitamin B12 supply because of the overall very low intake of vitamin B12 from fortified foods in this diet group (Fig. 1).

**Table 5:** Contribution from meat and dairy alternatives to total intake (without supplements) of energy, and qualitative aspects of the diet (protein, saturated fatty acids, sugar, fiber and micronutrients) in first-year students.

	Total (n = 142)	Vegans (n = 32)	Vegetarians (n = 66)	Omnivores (n = 44)	p-value*
Energy %	7.7 (2.7–11.3)	11.9^a^ (8.7–13.8)	6.6^b^ (1.6–10.2)	4.6^b^ (1.5–6.7)	< 0.001
Protein %	11.7 (2.6–19.2)	18.1^a^ (7.4–26.1)	10.1^b^ (2.9–15.9)	6.7^b^ (0.8–10.5)	< 0.001
SFA %	11.2 (1.3–15.4)	24.1^a^ (12.8–37.2)	7.3^b^ (0.8–9.4)	3.1^b^ (0.7–4.6)	< 0.001
Total sugar %	3.4 (0.2–4.1)	4.5^a^ (1.1–5.5)	3.5^b^ (0.2–2.9)	1.7^b^ (0.1–1.9)	0.002
Fiber %	8.6 (3.0–11.5)	11.4^a^ (5.3–17.1)	8.1^a,b^ (2.8–10.8)	6.1^b^ (1.9–7.8)	0.009
Vitamin B2%	10.1 (1.8–14.3)	15.6^a^ (5.4–24.8)	9.3^b^ (1.7–11.5)	4.5^b^ (0.7–8.0)	< 0.001
Vitamin B12%	16.5 (0.0–16.9)	44.1^a^ (0.0–87.1)	8.6^b^ (0.0–8.6)	1.5^b^ (0.0–0.0)	< 0.001
Folate %	8.8 (1.6–14.0)	13.4^a^ (4.6–20.0)	7.3^b^ (1.6–10.9)	6.1^b^ (1.0–12.4)	0.001
Iron %	10.8 (4.3–16.3)	13.8^a^ (5.9–21.6)	10.3^a,b^ (3.6–14.9)	8.1^b^ (1.6–11.8)	0.025
Zink %	8.3 (2.3–13.2)	11.4^a^ (5.5–16.4)	8.1^a,b^ (2.1–13.8)	4.5^b^ (0.8–7.7)	0.002
Calcium %	12.8 (2.8–19.8)	21.5^a^ (8.2–29.5)	10.1^b^ (2.3–15.4)	7.4^b^ (1.4–11.5)	< 0.001

Data presented as median (interquartile range); *p-values refer to differences between diet groups using Kruskal–Wallis test with Bonferroni post-hoc test; ^a,b^diet groups not sharing a common superscript letter are significantly different, p < 0.05; SFA, saturated fatty acids.

## Discussion

UPF constituted 49% of the daily energy intake, irrespective of the plant-forward eating pattern. Qualitative analysis of the diet revealed that UPF contributed to a significant portion of nutrient intake across all diet groups. However, an assessment of the qualitative aspects of the consumed foods revealed that UPF exhibited lower content of protein, fiber, vitamin B2, vitamin B12, folate, zinc, and calcium compared to processed foods (NOVA 3). The subgroup of plant-based alternatives had a lower content of SFA, a higher fiber content, and a lower energy density than consumed animal-based products. Conversely, plant-based alternatives exhibited lower protein content and a lower content of certain micronutrients.

### Diet quality and nutritional status in omnivores, vegetarians and vegans

Vegans and vegetarians exhibited lower protein, higher fiber, and a trend towards a lower energy intake compared to omnivores (Table 2). These characteristics are consistent with those observed in previous large-scale cohort studies^21,22^ and in recent data from Germany^23,24^. Overall, the total macronutrient intake was generally aligned with the recommendations of the German Society of Nutrition, while only the intake of SFA and total sugar exceeded the recommended levels^18^. Most micronutrients assessed (vitamin B12, folate, iron, zinc, and calcium) were on average consumed at levels below the recommended intake^18^.

We found an equally high UPF consumption in all diet groups, while other studies have indicated that a higher avoidance of animal-based foods is associated with a higher UPF consumption^10^. Since UPF are characterized as energy-dense and nutrient-poor^19^, they may aggravate the low intake of critical nutrients in vegans and vegetarians. By contrast, our data indicate a risk of undersupply with vitamin B12 in all diet groups (Fig. 1A). Due to the usual supplementation practice, no significant differences were observed in HTC levels between vegans and omnivores (Fig. 1B). Those results are in accordance with previous findings from Germany that also found no differences in HTC levels between vegans and omnivores^25^. However, vegetarians showed lower HTC levels, possibly because of lower supplementation rates of vitamin B12 compared to vegans. Vitamin B12 supplements were taken by 60% of all participants and 78% of vegans, which is consistent with other studies that have reported supplementation rates of 82–92% among German vegans^24–26^. Nevertheless, over 20% of vegans still lack vitamin B12 supplementation, underscoring the necessity for further education on this topic, particularly as plant-based diets continue to gain popularity across all age groups and educational levels. Notably, the prevalence of low HTC levels was similar across all diet groups and amounted to 16% even in omnivores. This unexpected finding may be attributed to a reduction in the bioavailability of vitamin B12 in UPF like processed meat products^27^. This conundrum requires further investigation, as vitamin B12 deficiency in women of childbearing age is clinically significant^28^.

Even though vegans demonstrated a higher iron intake than vegetarians and omnivores (Fig. 1C), the ferritin levels across the different diet groups were comparable (Fig. 1D). This is likely attributed to the reduced bioavailability of non-heme iron and inhibitors such as phytic acid and polyphenols in plant-based foods^29^. It can be reasonably inferred that iron intake should be approximately 1.8-times higher than that observed in omnivorous diets^29^.

### Contribution of UPF to diet quality

The lower content of protein, fiber, vitamin B2, vitamin B12, folate, zinc, and calcium of UPF compared to processed foods (Table 3, Fig. 2) are supported by findings from market analyses that have shown a poorer nutritional profile of UPF compared to less processed foods^30^. Such a poor nutritional profile of UPF has been identified as a main driver of their detrimental impact on health (for review, see^31^). Although UPF account for 46% of total protein intake, the protein content of UPF is lower (13%) than that of processed foods (19%). The lower protein intake with high consumption of UPF has been shown to be associated with a higher total energy intake, while the absolute protein intake remained relatively constant^32^. The low protein content together with a low fiber and high sugar content and a higher energy density, may thus explain the elevated risk of overweight and obesity associated with a high UPF consumption^33^. The difference in the mean energy density of 2.88 kcal/g in UPF and 1.95 kcal/g in NOVA3 foods may, however, not be relevant because the relationship between energy density and caloric intake was found to be non-linear, with consumption only increasing up to an energy density of approximately 1.5 kcal/g and slightly decreasing thereafter^34^.

Our data demonstrate that UPF contain lower amounts of vitamin B2, vitamin B12, calcium, and zinc compared to processed foods (Fig. 2). These results add to the finding that high UPF consumption is associated with a lower micronutrient intake^35^. The iron content was unexpectedly higher in UPF than in processed foods, presumably due to the high iron content in ultra-processed meat products and cocoa or soy-containing UPF. The latter advantage is, however, compensated by a reduced bioavailability of non-heme iron in plant-based products^29^. Accordingly, the substitution of UPF with less processed foods is expected to result in an enhancement of overall diet quality. Replacing UPF with less processed alternatives was predicted to reduce health implications due to the lower nutritional quality of UPF^7^. The authors therefore concluded that the availability of information on the degree of processing, which is currently lacking for consumers, could enhance population health.

### Contribution of plant-based alternatives to diet quality

In all diet groups, plant-based alternatives were consumed, with the highest prevalence among vegans, who consumed these products on a daily basis (Table 5). Despite comprising merely 12% of energy intake among vegans, these products provided 18% of protein intake but also 24% of SFA intake. Not all plant-based alternatives were classified as UPF (51% of dairy and 89% of meat alternatives), which is noteworthy, given the observed association between plant-based non-UPF and reduced cardiovascular risk, and the observed positive association between plant-based UPF and cardiovascular risk^16^. The underlying mechanisms for the adverse effects of plant-based UPF remain unclear. The lower SFA content and lower energy density of plant-based alternatives (Fig. 3) compared to animal products are unlikely to be the cause as they even represent an advantage of these products^36,37^. The higher fiber content in plant-based alternatives may also be perceived as favorable but the quantities are mostly inadequate to qualify as a substantial source of fiber^38^. For example, dairy alternatives contained only 0.9g of fiber per 100g. The dietary fibers employed for technological purposes in UPF are often synthetic or isolated, which may not provide the same micronutrients or health benefits as those derived from whole foods^39^. The impact of these fibers on a food matrix that may lower the eating rate and thus energy intake remains uncertain^40^. A disadvantage of plant-based alternatives is the lower protein content compared with animal-based products (Fig. 3). Although protein intake was adequate in all diet groups, a higher protein intake could promote satiety and thus prevent overconsumption (for review, see^41^).

In terms of micronutrients, plant-based dairy alternatives were generally less adequate than plant-based meat alternatives because they contain lower amounts of nutrients compared to animal-based dairy products, except for iron. By contrast, plant-based meat alternatives showed a higher content of iron, calcium, and folate than the consumed meat products (Table 4). These findings are consistent with previous research conducted in Germany, suggesting that plant-based meat alternatives could contribute to daily iron requirements^42^. In addition, the plant-based meat alternatives, rather than the plant-based dairy alternatives, serve as a source of calcium. This is presumably attributed to calcium-rich ingredients derived from soy and other legumes. In light of the low calcium intake in all diet groups (Table 2) and the growing consumption of these products in Germany^11^, the low calcium content represents a significant public health concern, particularly among vulnerable populations, including adolescents and adults who need to increase their peak bone mass. Fortification is, therefore, a good option for making these plant-based alternatives a viable source of not only calcium but also vitamin B12. However, other authors take a critical view of UPF fortification, making the food appear more positive^43^.

### Strength and limitations

Accurate dietary assessment was obtained by following the best practice approach for NOVA classification^20^ with a sensitivity analysis for quantification of uncertainty to increase the comparability of the estimates (see results). A key strength of our study is the thorough documentation of the brands of consumed foods, enabling detailed analysis of ingredients and therefore the classification according to NOVA. However, due to missing information on the recipes, the amounts of ingredients were adjusted to match the nutritional information on the product. For missing information on novel ingredients (e.g., bamboo fiber) that were not included in the BLS database assumptions based on existing literature were used.

In addition, evaluating consumption based on 3 days does not allow for the detection of occasionally consumed products. The lack of significant correlations between nutrient intake and parameters of nutritional status is likely explained by the use of dietary supplements, but in the case of ferritin, may also be due to the collection of dietary data within two weeks of blood sampling. As the dose of supplements taken was not recorded, this could not be considered in the nutrient intake.

In conclusion, our findings indicate an inadequate intake of many micronutrients by young adults in Germany. The lower content of micronutrients in UPF is a contributing factor to this phenomenon. Substituting UPF with processed foods could, therefore, contribute to an improvement in diet quality. The problem of insufficient micronutrient intake is further aggravated by a plant-forward eating pattern. This is also reflected in the lower micronutrient content of plant-based alternatives. A fortification of plant-based milk alternatives could, therefore, contribute to an improvement in diet quality.

## Data Availability

The data supporting this study’s findings are not publicly available due to the data privacy statement in the subject information form and are available from the corresponding author A.B.-W. upon reasonable request.
